# Portal Vein Stenting Combined with ^125^I Particle Chain Implantation Followed by As_2_O_3_ in the Treatment of Hepatocellular Carcinoma with Portal Vein Tumour Thrombus

**DOI:** 10.1155/2020/4109216

**Published:** 2020-01-31

**Authors:** Zhaonan Li, Guangyan Si, De-Chao Jiao, Xinwei Han, Wenguang Zhang, Yahua Li, Xueliang Zhou, Juanfang Liu, Jianjian Chen

**Affiliations:** ^1^Department of Interventional Radiology, First Affiliated Hospital of Zhengzhou University, Zhengzhou 450000, China; ^2^Department of Interventional Radiology, The Affiliated Hospital of Traditional Chinese Medicine, Southwest Medical University, Luzhou 646000, China

## Abstract

**Objective:**

To evaluate the feasibility and safety of portal vein stenting (PVS) combined with ^125^I particle chain implantation and sequential arsenic trioxide (As_2_O_3_) for the treatment of hepatocellular carcinoma (HCC) with portal vein tumour thrombus (PVTT) by transcatheter arterial chemoembolization (TACE).

**Methods:**

From January 2015 to January 2018, the clinical data of 30 patients with HCC complicated by PVTT were retrospectively analysed (26 men and 4 women). The laboratory examinations, incidence of adverse events, cumulative survival rate, and stent patency were analysed for all enrolled patients.

**Results:**

The success rate of interventional treatment in all patients was 100%. The results of the laboratory tests before and 1 week after surgery showed that the mean concentrations of alanine aminotransferase (ALT) and aspartate aminotransferase (AST) decreased from 50.9 U/L ± 25.8 to 41.8 U/L ± 21.6 (*P* < 0.001) and 57.6 U/L ± 19.9 to 44.2 U/L ± 26.1 (*P* < 0.001) and 57.6 U/L ± 19.9 to 44.2 U/L ± 26.1 (

**Conclusion:**

PVS combined with ^125^I particle chain implantation followed by TACE with As_2_O_3_ is safe and feasible for patients with PVTT. The long-term efficacy of this treatment needs to be further studied.

## 1. Introduction

Patients with hepatocellular carcinoma (HCC) are highly susceptible to invasion of the portal vein, which forms portal vein tumour thrombus (PVTT) [1, 2]. PVTT can cause partial or total portal vein occlusion and extensive intra- or extrahepatic metastases. Moreover, PVTT in the main portal trunk exerts pressure on the portal vein, which further leads to gastrointestinal bleeding and ascites and induces intrahepatic tumour dissemination and recurrence. If effective treatment is not available, the median survival time of these patients is only 2.7–4.0 months [3]. In recent years, portal vein stenting (PVS) combined with ^125^I implantation has achieved significant effects in treating main portal vein tumour thrombus [4–7]. Stent implantation in the main portal vein can effectively alleviate portal vein pressure, relieve clinical symptoms, and improve quality of life [8].

In 2004, arsenic trioxide (As_2_O_3_) was approved for the treatment of human primary HCC by the State Food and Drug Administration of China. However, the single drug As_2_O_3_ does not benefit patients diagnosed with solid tumours. The therapeutic benefit arises when this drug is combined with transcatheter arterial chemoembolization (TACE) [9]. In the rabbit HCC VX2 model, Kim et al. reported that a combination of TACE with As_2_O_3_-iodized oil emulsion had potent anticancer effects without a significant increase in hepatic and renal toxicity [10]. In the same model, Yu et al. demonstrated the efficacy of As_2_O_3_ nanoparticles in combination with arterial embolization hyperthermia [11].

Therefore, this study aimed to further investigate the efficacy of portal vein stenting combined with ^125^I particle chain implantation followed by As_2_O_3_ in the treatment of hepatocellular carcinoma with portal vein tumour thrombus. The findings can provide new clues to the best treatments for these patients.

## 2. Materials and Methods

### 2.1. Study Design

This retrospective study was conducted between January 2015 and January 2018 at the First Affiliated Hospital of Zhengzhou University, China. This study aimed to evaluate the therapeutic value of PVS combined with ^125^I particle chain endovascular implantation followed by TACE together with As_2_O_3_ in treating patients with HCC complicated by PVTT. This study was approved by the Ethics Committee of the First Affiliated Hospital of Zhengzhou University. The need for individual consent was waived by the committee because of the retrospective nature of the study. The principles of the Declaration of Helsinki and Good Clinical Practice Guidelines were strictly followed.

### 2.2. Patient Selection

Thirty patients with advanced HCC complicated by PVTT were enrolled in this study. There were 26 men and 4 women with a median age of 57 years (range, 45–76). The inclusion criteria were as follows: (1) clinical diagnosis of HCC with PVTT (established by history, tumour markers, hepatitis series, imaging, and/or pathology); (2) the target lesion had at least one diameter line available for measurement; (3) Child-Pugh class A or B; (4) no previous systemic treatment, such as oral molecularly targeted drugs or systemic chemotherapy; (5) informed clinical consent was provided for the treatment; and (6) adequate renal function (defined as serum creatinine ≤1.5 × the upper limit of normal). The exclusion criteria were as follows: (1) age older than 80 years; (2) Child-Pugh class C or D; (3) life expectancy <3 months; (4) coagulation disorders that could not be corrected; (5) TACE contraindications: severe cardiopulmonary liver and kidney dysfunction, high-flow hepatic artery. Portal shunt or hepatic artery-hepatic vein shunt, blood system disease or coagulopathy; (6) unable to cooperate with treatment and the observer due to various factors; (7) widespread metastases; or (8) massive ascites.

### 2.3. Treatment Process

After local anaesthesia, the patient's 2^nd^ order branch of the intrahepatic portal vein was punctured with a 22G Chiba needle (Cook Medical, Bloomington, IN, USA) under ultrasound guidance. Then, the 5F catheter sheath (Cook Medical, Bloomington, IN, USA) was exchanged, and the portal vein or superior mesenteric vein was imaged by a pig tail catheter (Cordis, USA), and the length and diameter of the obstructed section of the main portal vein was measured. The number of ^125^I seeds = length of obstructed MPV (mm)/4.5 + 2. The implanted ^125^I particles were confirmed to produce enough radiation energy to completely cover the PVTT segment. These ^125^I seeds were arranged linearly and sealed into a 4F sterile plastic tube to construct the ^125^I seed strand. Then, two 0.035-inch diameter hard guide wires (Terumo, Tokyo, Japan) were inserted into the splenic vein. Next, a stent of a suitable size (Bard, USA) was placed along the guide wire and released in the obstructed segment, and the other guide wire was again fed into the 5F catheter sheath to the obstructed section of the main portal vein. When the 5F catheter sheath was withdrawn, the radioactive seed strand was released and fixed steadily between the stent and MPV. Portography was performed again. Finally, the transhepatic puncture track was occluded by coils (Cook Medical, Bloomington, IN, USA) with diameters of 3–5 mm.

### 2.4. Arsenic Trioxide Transarterial Chemoembolization

All TACE procedures were performed by three interventionists with 10 years of experience in interventional radiology. As_2_O_3_ (20 mg) was diluted in 0.9% NaCl, mixed with a maximum of 20 mL iodized oil (lipiodol) per session, and injected through a 2.7–3.0-F microcatheter for vessel occlusion (Qian [12]; Hu [13]). If necessary, embolization particles such as PVA particles or microspheres were used to strengthen embolization. The embolization extent was determined according to the tumour size and the patients' liver function.

### 2.5. Quantitative Evaluation

All patients received supportive liver protection therapy for at least 3 days, and intravenous nutrition drugs were appropriately added. The change in tumour size was determined from radiological evaluations using CT. According to the Modified Response Evaluation Criteria in Solid Tumours (RECIST) [14, 15], intrahepatic tumour response was classified into one of four categories: complete response (CR), partial response (PR), stable disease (SD), and progressive disease (PD). The modified standard was used for PVTT evaluation [16]. The categories were as follows: (i) CR, thrombus disappearance and restored the PV; (ii) PR, >50% reduction of thrombus in the greatest cross-sectional area; (iii) SD, <50% reduction or <25% increase; and (iv) PD, >25% increase or MPV invasion. The definition of the DCR for PVTT was the same as that for intrahepatic tumours. Adverse events (AEs) were measured as the secondary end points. AEs were graded according to the Common Toxicity Criteria for Adverse Events (CTCAE) version 4.0 [17]. The primary end point was OS, which was determined as the period from the day of the initial procedure until death.

### 2.6. Follow-Up

Treatment-related adverse reactions were recorded in the first week after treatment, and tumour response was assessed by RECIST 2 months after the operation. Biochemical examinations (blood routine, coagulation function, and liver and kidney function), performance status, clinical signs, and imaging examinations (colour Doppler ultrasound, computed tomography, or single-photon emission computed tomography) were performed monthly.

## 3. Statistical Method

Continuous data are presented as the mean ± SD. Quantitative variables before and after stent placement were compared using the paired sample *t*-test. The cumulative survival rate and cumulative stent patency were estimated with Kaplan–Meier survival analysis using SPSS version 22.0 (SPSS Inc., Chicago, IL). *P* < 0.05 was defined as statistically significant.

## 4. Result

### 4.1. Characteristics of the Patients

(Table 1) presents the characteristics of the 30 patients included. The mean age was 54 years (age range, 44–76). The mean number of ^125^I seeds loaded was 17.8 ± 5.2 (range, 11–26). The mean intended dose was 62.6 ± 1.8 Gy (range, 58.3–64.0 Gy). The Child-Pugh classification was A in 12 patients and B in eighteen patients. Sixteen patients had multiple scattered lesions, and fourteen had a single lesion. Six patients had right portal vein tumour thrombus, four had left portal vein tumour thrombus, eight had main and right portal vein tumour thrombus, and twelve had main and left portal vein tumour thrombus.

### 4.2. Safety and Complications

A 100% technical success rate was achieved in all patients. The results of the laboratory tests before and 1 week after intervention showed that the mean concentrations of alanine aminotransferase (ALT) and aspartate aminotransferase (AST) decreased from 50.9 U/L ± 25.8 to 41.8 U/L ± 21.6 (*P* < 0.001) and 57.6 U/L ± 19.9 to 44.2 U/L ± 26.1 (*P* < 0.001), respectively. There was no significant difference in the other laboratory test results (Table 2). Treatment-related adverse events occurred in 14 of the 30 patients. As shown in (Table 3), no grade 3 adverse events were observed. The adverse events included fever, haemorrhage, abdominal pain, and leukopenia, which occurred in 7/30 (23.3%), 3/30 (6.7%), 3/30 (10.0%), and 1/30 (3.3%) patients, respectively. Of all the adverse events, 3 patients (10.0%) had a transient fever with a temperature >38.5°C, which needed to be controlled with symptomatic therapy. Two patients (6.7%) needed dezocine (Yangtze River Pharmaceutical Industry Co., Ltd., Taizhou, China) for abdominal pain. One patient (3.3%) developed leukocytopenia and recovered after treatment with recombinant human granulocyte colony-stimulating factor (China Jinan Qilu Pharmaceutical Co., Ltd.). No stent or ^125^I seed migration was detected during follow-up.

### 4.3. Response of Hepatocellular Carcinoma and Tumour Thrombus

After 2 months of treatment, according to the RECIST, 0% (0/30), 26.7% (8/30), 53.3% (16/30), and 20% (6/30) of the patients with HCC achieved CR, PR, SD, and PD after chemoembolization, respectively. Moreover, the disease control rate (SD + PR + CR) of intrahepatic tumours was 80.0% (24/30). CR, PR, SD, and PD were observed in 0% (0/30), 20.0% (6/30), 63.3% (19/30), and 16.7% (5/30) of the patients with PVTT, respectively, while the disease control rate (SD + PR + CR) of tumour thrombus was 83.3% (25/30) (Table 4).

### 4.4. Survival and Stent Patency

**Figure 1:**
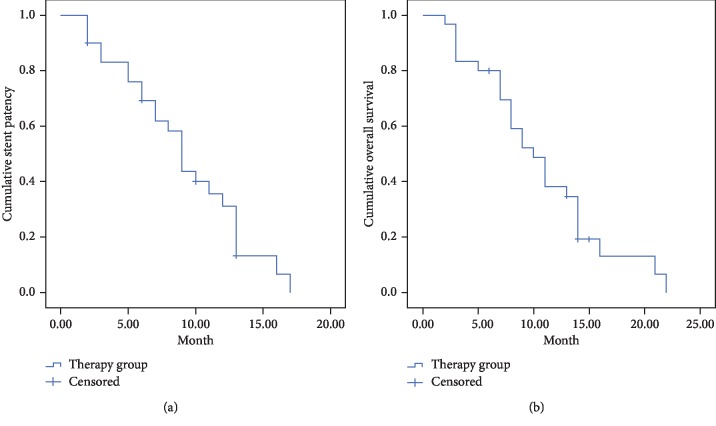
Cumulative stent patency and Kaplan–Meier overall survival curves. (a) The mean stent patency period was 8.4 ± 4.2 months (range, 2–17 months). The 3-, 6-, 9-, and 12-month cumulative stent patency rates were 83.1%, 69.2%, 43.7%, and 31.2%, respectively. (b) The mean survival was 10.0 ± 5.1 months (range, 2–22 months). The 3-, 6-, 9-, and 12-month cumulative survival rates were 83.3%, 80.0%, 52.2%, and 38.3%, respectively.

The follow-up period ended in January 2018. The mean survival time was 10.0 ± 5.1 months (range, 2–22 months). The cumulative survival rates at 3, 6, 9, and 12 months were 83.1%, 69.2%, 43.7%, and 31.2%, respectively, while the patency rates of the stents were 83.3%, 80.0%, 52.2%, and 38.3%, respectively (Figure 1). Of the 30 deaths during the follow-up period, 16 patients died of liver failure, 8 died of gastrointestinal bleeding, 3 died of lung metastasis, 2 died of brain metastases, and 1 died of heart failure because the tumour invaded the atria.

## 5. Discussion

Currently, little data are available about the combined therapeutic strategy of As_2_O_3_ and TACE combined with endovascular implantation of ^125^I seeds for patients with HCC and PVTT [18, 19]. Therefore, this study was conducted to further explore the treatment efficacy of As_2_O_3_ combined with endovascular implantation of ^125^I particle chain followed by As_2_O_3_ for patients with HCC and PVTT. The results showed that the technical success rate of the treatment was 100%.

A portal vein tumour thrombus blocks portal vein blood perfusion, which impairs liver function and easily leads to liver failure or death. Therefore, it is often considered a contraindication for the implementation of TACE; thus, opening the portal vein trunk and restoring blood flow is a guarantee for the safe implementation of TACE. In 1999, Japanese scholar Yamakado et al. first used PVS combined with TACE to treat patients with HCC and PVTT, which led to significant benefits [20]. Zhang et al. found that the survival rate of patients with a portal vein stent combined with TACE was significantly higher than that of patients treated with TACE alone [21]. In 2001, Yamakado et al. suggested that there was no vascular endothelium coverage on the surface after 16 months of stent implantation in the portal cavity, indicating that endothelial proliferation is not the main cause of stent obstruction [22]. Therefore, effective treatment of the tumour thrombus and patency of the stent can be guaranteed. However, because the portal vein stent is only open to the blocked portal vein and has no therapeutic effect on the tumour thrombus, the stent may still be occluded as the tumour thrombus develops.

With the application of the interstitial implantation of radioactive particles in the treatment of various solid tumours, ^125^I seeds were also tested for HCC and PVTT and achieved favourable results. The 0.5-, 1-, and 2-year survival rates of the observation group were superior to those of the control group [23]. ^125^I seeds have a half-life of 59.6 d and a radiation diameter of 1.7 cm. This special property can release continuous low doses of *x*- and *γ*-rays, leading to a total dose of 160–180 Gy to the local tissue. The principle of treatment is that *γ*-rays can cause double-stranded and single-strand DNA breaks in the cancer cells, which causes the cancer cells to be damaged, and the particles emit ionized water molecules to cause ionization, resulting in DNA damage. Some studies have shown that *β*- or *γ*-rays can inhibit neointimal hyperplasia. Zhang et al. retrospectively analysed the combination of TACE and portal vein stents with or without intravascular ^125^I seed strips for the treatment of HCC with PVTT. The results showed that portal stent and TACE combined with ^125^I particle strips significantly prolonged survival and stent patency [24]. The results of Chuan-Xing et al. [25] are similar to those of Zhang et al. Moreover, Fang et al. confirmed this point in a randomized controlled study [26].

Serum VEGF is significantly increased after TACE, and VEGF mediates HCC angiogenesis, portal vein thrombosis formation, and its evolution. Sorafenib is currently approved as the only systemic therapy for PHC by the American Food and Drug Administration and inhibits angiogenesis by targeting the vascular endothelial growth factor receptor 2 (VEGFR2) and platelet-derived growth factor receptor (PDGFR) pathways [27–29]. In patients with advanced HCC, TACE combined with sorafenib prolonged overall survival (OS) significantly [30–33]. However, most patients with HCC in China cannot afford such treatment, and some choose As_2_O_3_ as a replacement therapy for sorafenib. As_2_O_3_ can induce apoptosis and inhibit the proliferation of hepatocarcinoma cells, reduce telomerase activity, downregulate VEGF expression, and improve cellular immune function [34–36]. Li et al. found that TACE combined with As_2_O_3_ could induce the apoptosis of carcinoma cells, and the tumour apoptosis‐inhibitory protein survivin might have played a significant role [37]. In Hu's study, TACE with As_2_O_3_, a more cost‐effective treatment with a good clinical benefit rate of 60%, had a lower risk and better response than TACE plus sorafenib or S‐1 plus *α*-interferon [38]. Moreover, there was a statistically significant difference in mOS between the treatment group and the control group.

This study discusses PVS combined with ^125^I particle chain implantation followed by As_2_O_3_ in the treatment of HCC with PVTT. The results of the laboratory tests before and 1 week after treatment showed that the mean concentrations of alanine aminotransferase and aspartate aminotransferase decreased from 50.9 U/L ± 25.8 to 41.8 U/L ± 21.6 (*P* < 0.001) and 57.6 U/L ± 19.9 to 44.2 U/L ± 26.1 (*P* < 0.001), respectively. No complications in grade 3 or higher according to the Common Terminology Criteria for Adverse Events were observed. However, there are still some limitations in this study. First, the small sample size of the study did not allow for a detailed analysis of the factors affecting the prognosis of the portal vein stent combined with ^125^I seed strip and sequential As_2_O_3_. In addition, the retrospective nature of the study prevented an assessment of factors that were not routinely collected. Finally, this study failed to compare the results of this treatment with a control group, such as sorafenib + TACE, and additional studies are necessary to adequately assess the use of this treatment approach.

## Figures and Tables

**Table 1:** Characteristics of patients.

Characteristics	
Age, years, median (range)	57 (45–76)

Sex (male/female)	26/4

Number of lesions	
Single	14 (47%)
Multiple	16 (53%)

Location of tumour thrombosis	
LPVB	4 (13%)
RPVB	6 (20%)
MPV + LPVB	12 (40%)
MPV + RPVB	8 (27%)

Child-pugh class	
A	12 (40%)
B	18 (60%)

Degree of PVTT, *n* (%)	
Vp2	6 (20%)
Vp3	14 (47%)
Vp4	10 (33%)

The Liver Cancer Study Group of Japan suggested a macroscopic classification for PVTT, which categorized PVTT into five grades: (1) Vp0, no PVTT; (2) Vp1, presence of PVTT not in, but distal to, the 2^nd^ order branches of the portal vein; (3) Vp2, presence of PVTT in the 2^nd^ order branches of the portal vein; (4) Vp3, presence of PVTT in the 1^st^ order branches of the portal vein; and (5) Vp4, presence of PVTT in the main trunk of the portal vein or a portal vein branch contralateral to the mainly involved lobe (or both). LPVB: left portal vein branch; RPVB: right portal vein branch; MPV: main portal vein; PVTT: portal vein tumour thrombus.

**Table 2:** Results of laboratory tests before and 1 week after procedure.

Laboratory test	Before procedure^*∗*^	1 week after procedure^*∗*^	*P* value
TB (*μ*mol/L)	14.0 ± 4.5	16.3 ± 7.2	0.427
ALB (g/L)	37.5 ± 9.4	39.2 ± 5.1	0.612
ALT (U/L)	50.9 ± 25.8	41.8 ± 21.6	0.001
AST (U/L)	57.6 ± 19.9	44.2 ± 26.1	0.001
CR (*μ*mol/L)	75.3 ± 15.3	71.9 ± 10.2	0.329
PT (s)	11.9 ± 2.4	14.2 ± 3.2	0.149
WBC (10^9^/L)	6.1 ± 2.6	5.3 ± 1.8	0.347
PLT (10^9^/L)	143.6 ± 64.6	151.0 ± 82.3	0.378

ALB = albumin; ALT = alanine transaminase; AST = aspartate transaminase; CR = creatinine; CTCAE = Common Toxicity Criteria for Adverse Events; PLT = platelets; PT = prothrombin time; TB = total bilirubin. ^*∗*^Data expressed as mean ± SD.

**Table 3:** Adverse events related to procedure.

Adverse event	All events	Grade of toxicity CTCAE		
		1	2	≥3
Fever	7 (23.3%)	4 (13.3%)	3 (10%)	0
Hemorrhage	3 (10%)	2 (6.7%)	1 (3.3%)	0
Stent or seeds migration	0	0	0	0
Abdominal pain	3 (10%)	1 (3.3%)	2 (6.7%)	0
RILD	0	0	0	0
Leukopenia	1 (3.3%)	1 (3.3%)	0	0
Gastrointestinal ulceration	0	0	0	0
Total	14 (46.7%)	7 (23.3%)	6 (20%)	0

Note: data are number of patients (%); RILD = radiation-induced liver disease. Adverse events were assessed after 1 week of procedure.

**Table 4:** Tumour response.

Tumour response	Intrahepatic tumour	Tumour thrombus
CR	0	0
PR	8 (26.7%)	6 (20%)
SD	16 (53.3%)	19 (63.3%)
PD	6 (20%)	5 (16.7%)
DCR^*∗*^ (%)	80	83.3

CR = complete response; DCR = disease control rate; PD = progressive disease; PR = partial response; SD = stable disease. ^*∗*^DCR = (SD + PR + CR)/*n*.

## Data Availability

The clinical data were obtained from the interventional department of the First Affiliated Hospital of Zhengzhou University. The data used to support the findings of this study are available from the corresponding author upon request.

## References

[1] Luo F., Liao R. (2019). Hepatectomy for hepatocellular carcinoma patients with portal vein tumor thrombus: benefit or not. *Hepatology*.

[2] Yuan D., Gao Z., Zhao J., Zhang H., Wang J. (2019). ^125^I seed implantation for hepatocellular carcinoma with portal vein tumor thrombus: a systematic review and meta-analysis. *Brachytherapy*.

[3] Villa E., Moles A., Ferretti I. (2000). Natural history of inoperable hepatocellular carcinoma: estrogen receptors’ status in the tumor is the strongest prognostic factor for survival. *Hepatology*.

[4] Yu T. Z., Zhang W., Liu Q. X. (2017). Endovascular brachytherapy combined with portal vein stenting and transarterial chemoembolization improves overall survival of hepatocellular carcinoma patients with main portal vein tumor thrombus. *Oncotarget*.

[5] Sun J. H., Zhou T., Zhu T. (2016). Portal vein stenting combined with iodine-125 seeds endovascular implantation followed by transcatheter arterial chemoembolization for treatment of hepatocellular carcinoma patients with portal vein tumor thrombus. *Biomed Research International*.

[6] Zhang X.-B., Wang J.-H., Yan Z.-P., Qian S., Du S.-S., Zeng Z.-C. (2009). Hepatocellular carcinoma with main portal vein tumor thrombus. *Cancer*.

[7] Higaki I., Hirohashi K., Kubo S. (2000). Portal vein stenting to treat portal vein tumor thrombus in hepatocellular carcinoma. *Osaka City Medical Journal*.

[8] Lu J., Guo J.-H., Zhu H.-D., Zhu G.-Y., Chen L., Teng G.-J. (2017). Safety and efficacy of irradiation stent placement for malignant portal vein thrombus combined with transarterial chemoembolization for hepatocellular carcinoma: a single-center experience. *Journal of Vascular and Interventional Radiology*.

[9] Subbarayan P. R., Ardalan B. (2014). In the war against solid tumors arsenic trioxide need partners. *Journal of Gastrointestinal Cancer*.

[10] Kim H. J., Shin J. H., Kim T.-H. (2009). Efficacy of transarterial embolization with arsenic trioxide oil emulsion in a rabbit VX2 liver tumor model. *Journal of Vascular and Interventional Radiology*.

[11] Yu H., Zhu G. Y., Xu R. Z. (2011). Arterial embolization hyperthermia using As2O3 nanoparticles in VX2 carcinoma-induced liver tumors. *PLoS One*.

[12] Qian J. (2011). Interventional therapies of unresectable liver metastases. *Journal of Cancer Research and Clinical Oncology*.

[13] Hu H. T., Yao Q. J., Meng Y. L. (2017). Arsenic trioxide intravenous infusion combined with transcatheter arterial chemoembolization for the treatment of hepatocellular carcinoma with pulmonary metastasis: long-term outcome analysis. *Journal of Gastroenterology and Hepatology*.

[14] Forner A., Ayuso C., Varela M. (2009). Evaluation of tumor response after locoregional therapies in hepatocellular carcinoma. *Cancer*.

[15] Barnacle A. M., McHugh K. (2006). Limitations with the response evaluation criteria in solid tumors (RECIST) guidance in disseminated pediatric malignancy. *Pediatric Blood & Cancer*.

[16] Kang J., Nie Q., DU R. (2014). Stereotactic body radiotherapy combined with transarterial chemoembolization for hepatocellular carcinoma with portal vein tumor thrombosis. *Molecular and Clinical Oncology*.

[17] Liu Y.-J., Zhu G.-P., Guan X.-Y. (2012). Comparison of the NCI-CTCAE version 4.0 and version 3.0 in assessing chemoradiation-induced oral mucositis for locally advanced nasopharyngeal carcinoma. *Oral Oncology*.

[18] Song P., Hai Y., Ma W. (2018). Arsenic trioxide combined with transarterial chemoembolization for unresectable primary hepatic carcinoma. *Medicine*.

[19] He L., Xu Q., Chen L., Chen R. (2016). A meta-analysis of arsenic trioxide combined with transcatheter arterial chemoembolization for treatment of primary hepatic carcinoma. *Evidence-Based Complementary and Alternative Medicine*.

[20] Yamakado K., Tanaka N., Nakatsuka A., Matsumura K., Takase K., Takeda K. (1999). Clinical efficacy of portal vein stent placement in patients with hepatocellular carcinoma invading the main portal vein. *Journal of Hepatology*.

[21] Zhang X.-B., Wang J.-H., Yan Z.-P., Qian S., Liu R. (2009). Hepatocellular carcinoma invading the main portal vein: treatment with transcatheter arterial chemoembolization and portal vein stenting. *CardioVascular and Interventional Radiology*.

[22] Yamakado K., Nakatsuka A., Tanaka N. (2001). Portal venous stent placement in patients with pancreatic and biliary neoplasms invading portal veins and causing portal hypertension: initial experience. *Radiology*.

[23] Luo J.-J., Zhang Z.-H., Liu Q.-X., Zhang W., Wang J.-H., Yan Z.-P. (2016). Endovascular brachytherapy combined with stent placement and TACE for treatment of HCC with main portal vein tumor thrombus. *Hepatology International*.

[24] Jiang Z.-B., Shan H., Shen X. Y. (2004). Transjugular intrahepatic portosystemic shunt for palliative treatment of portal hypertension secondary to portal vein tumor thrombosis. *World Journal of Gastroenterology*.

[25] Chuan-Xing L., Xu H., Bao-Shan H. (2011). Efficacy of therapy for hepatocellular carcinoma with portal vein tumor thrombus. *Cancer Biology & Therapy*.

[26] Fang Z. T., Yan Z. P., Luo J. J. (2013). Evaluation of endovascular placement of iodine-125 seed strand combined with transcatheter arterial chemoembolization for treating hepatocellular carcinoma with extensive portal vein tumor thrombus. *Zhonghua Gan Zang Bing Za Zhi*.

[27] Iezzi R., Pompili M., Annicchiarico E. (2019). TACE with degradable starch microspheres (DSM-TACE) as second-line treatment in HCC patients dismissing or ineligible for sorafenib. *European Radiology*.

[28] Chen L., Zheng Y., Zhang H. (2018). Comparative analysis of tumor-associated vascular changes following TACE alone or in combination with sorafenib treatment in HCC: a retrospective study. *Oncology Letters*.

[29] Yao Q., Zhang H., Xiong B., Zheng C. (2017). Combination of sorafenib and TACE inhibits portal vein invasion for intermediate stage HCC: a single center retrospective controlled study. *Oncotarget*.

[30] Ren B., Wang W., Shen J., Li W., Ni C., Zhu X. (2019). Transarterial chemoembolization (TACE) combined with sorafenib versus TACE alone for unresectable hepatocellular carcinoma: a propensity score matching study. *Journal of Cancer*.

[31] Wang E., Xia D., Bai W. (2019). Tumor Hypervascularity and hand-foot-skin reaction predict better outcomes in combination treatment of TACE and Sorafenib for intermediate hepatocellular carcinoma. *BMC Cancer*.

[32] Geschwind J.-F., Kudo M., Marrero J. A. (2016). TACE treatment in patients with sorafenib-treated unresectable hepatocellular carcinoma in clinical Practice: final analysis of GIDEON. *Radiology*.

[33] Arizumi T., Ueshima K., Minami T. (2015). Effectiveness of sorafenib in patients with transcatheter arterial chemoembolization (TACE) refractory and intermediate-stage hepatocellular carcinoma. *Liver Cancer*.

[34] Sadaf N., Kumar N., Ali M., Ali V., Bimal S., Haque R. (2018). Arsenic trioxide induces apoptosis and inhibits the growth of human liver cancer cells. *Life Sciences*.

[35] Duan X., Li T., Han X. (2017). The antitumor effect of arsenic trioxide on hepatocellular carcinoma is enhanced by andrographolide. *Oncotarget*.

[36] Jiang L., Wang L., Chen L. (2015). As_2_O_3_ induces apoptosis in human hepatocellular carcinoma HepG2 cells through a ROS-mediated mitochondrial pathway and activation of caspases. *International Journal of Clinical and Experimental Medicine*.

[37] Li H., Gong J., Jiang X., Shao H. (2013). Arsenic trioxide treatment of rabbit liver VX-2 carcinoma via hepatic arterial cannulation-induced apoptosis and decreased levels of survivin in the tumor tissue. *Croatian Medical Journal*.

[38] Meng Y. L., Hu H. T., Li H. L. (2012). The clinical therapeutic effects of arsenic trioxide combined with transcatheter arterial chemoembolization in treating primary liver cancer with pulmonary metastases. *Zhonghua Nei Ke Za Zhi*.

